# RETRACTION: Analysis of Surgical Site Infection and Tumour‐Specific Survival Rate in Patients With Renal Cell Carcinoma After Laparoscopic Radical Nephrectomy

**DOI:** 10.1111/iwj.70617

**Published:** 2025-04-21

## Abstract

**RETRACTION:** MengS. , MengM. , WangS. , and ZhengW. , “Analysis of Surgical Site Infection and Tumour‐Specific Survival Rate in Patients With Renal Cell Carcinoma After Laparoscopic Radical Nephrectomy,” International Wound Journal 21, no. 5 (2024): e14711, 10.1111/iwj.14711.38387886 PMC10834101

The above article, published online on 01 February 2024, in Wiley Online Library (http://onlinelibrary.wiley.com/), has been retracted by agreement between the journal Editor in Chief, Professor Keith Harding; and John Wiley & Sons Ltd. Following an investigation by the publisher, all parties have concluded that this article was accepted solely on the basis of a compromised peer review process. In addition, further investigation by the publisher found that multiple included citations were irrelevant, which means large parts of the manuscript are unsubstantiated. The investigation also identified flaws and inconsistencies between the abstract and the results presented in the article. The editors have therefore decided to retract the article. The corresponding author could not be reached for comment regarding the retraction.

**RETRACTION:** S. Meng , M. Meng , S. Wang , and W. Zheng , “Analysis of Surgical Site Infection and Tumour‐Specific Survival Rate in Patients With Renal Cell Carcinoma After Laparoscopic Radical Nephrectomy,” International Wound Journal 21, no. 5 (2024): e14711, 10.1111/iwj.14711.38387886 PMC10834101

The above article, published online on 01 February 2024, in Wiley Online Library (http://onlinelibrary.wiley.com/), has been retracted by agreement between the journal Editor in Chief, Professor Keith Harding; and John Wiley & Sons Ltd. Following an investigation by the publisher, all parties have concluded that this article was accepted solely on the basis of a compromised peer review process. In addition, further investigation by the publisher found that multiple included citations were irrelevant, which means large parts of the manuscript are unsubstantiated. The investigation also identified flaws and inconsistencies between the abstract and the results presented in the article. The editors have therefore decided to retract the article. The corresponding author could not be reached for comment regarding the retraction.

